# Assessing the Nutritional and Neurodevelopmental Status in Children Attending Preschools in a Neighborhood in Bogotá, Colombia

**DOI:** 10.3390/nu18121996

**Published:** 2026-06-19

**Authors:** Laura Sofia Aguilera-Ariño, Claudia Talero-Gutiérrez, Alberto Velez-Van-Merbeeke, Natalia Pedraza-López, Maria Patiño-Rattiva, Isabella Pastrana-Bustamante, Juan Andrés Ospina-Arias, Mariana Quijano-Zauner, María José Velásquez, Sara Sofia Carvajal-Rincón, Angela María Pinzón-Rondón

**Affiliations:** 1 Neuroscience Research Group (Neuros), Centro de Neurociencia—Neurovitae, School of Medicine and Health Sciences, Universidad del Rosario, Carrera 24 #63c-69, Bogotá 111221, Colombia; lauras.aguilera@outlook.com (L.S.A.-A.); claudia.talero@urosario.edu.co (C.T.-G.); alberto.velez@urosario.edu.co (A.V.-V.-M.); 2 Clinical Research Group—Epidemiological Studies Line, School of Medicine and Health Sciences, Universidad del Rosario, Quinta de Mutis Campus, Carrera 24 #63c-69, Bogotá 111221, Colombia; natalia.pedraza@urosario.edu.co; 3 Undergraduate Neuroscience Research Group (Semineuros), Center of Neuroscience—Neurovitae, School of Medicine and Health Sciences, Universidad del Rosario, Carrera 24 #63c-69, Bogotá 111221, Colombia; mariap.patino@urosario.edu.co (M.P.-R.); isabella.pastrana@urosario.edu.co (I.P.-B.); juanan.ospina@urosario.edu.co (J.A.O.-A.); mariana.quijano@urosario.edu.co (M.Q.-Z.); mariajose.velasquez@urosario.edu.co (M.J.V.); saras.carvajal@urosario.edu.co (S.S.C.-R.)

**Keywords:** child development, neurodevelopmental disorders, nutritional status, malnutrition, anthropometry

## Abstract

**Background:** Early childhood nutrition is strongly associated with neurodevelopmental outcomes, particularly in socially vulnerable settings. Limited evidence is available describing the relationship between nutritional status, food security, and neurodevelopment among preschool children in low-income urban areas of Colombia. This study aimed to evaluate nutritional status, household food insecurity, and neurodevelopmental outcomes in children attending early childhood centers in El Codito, Bogotá, and to explore the association between anthropometric indicators and neurodevelopmental performance. **Methods:** A cross-sectional study was conducted in children enrolled in community childcare centers. Nutritional status was assessed using anthropometric indicators according to World Health Organization growth standards, including weight for age, height for age, and body mass index for age. Neurodevelopment was evaluated using the Escala Abreviada de Desarrollo (EAD). Household food insecurity was measured through a validated questionnaire. Descriptive statistics were performed, and associations between variables were analyzed using correlation tests and group comparisons according to data distribution. **Results:** Most participants presented adequate nutritional status; however, a proportion of children showed risk of stunting or excess weight. Neurodevelopmental scores were generally within expected ranges, although variability was observed across developmental domains. Significant associations were identified between certain anthropometric indicators and neurodevelopmental outcomes. Moderate to severe household food insecurity was identified in 21.4% of participating households. **Conclusions:** Nutritional status and household food insecurity represent important contextual factors for child health in vulnerable urban populations. These findings highlight the importance of integrated nutritional and developmental monitoring strategies within early childhood programs. Further longitudinal studies are required to clarify causal pathways and to guide targeted public health interventions in similar contexts.

## 1. Introduction

Child malnutrition is one of the main public health problems affecting low- and middle-income countries such as Colombia. It results from deficiencies, excesses, or imbalances in energy and nutrient intake and may manifest as acute malnutrition, stunting, overweight, or impairments in cognitive and neuromotor development [1,2]. Acute malnutrition is associated with increased morbidity and mortality, while stunting reflects the cumulative effects of chronic nutritional deprivation and has been linked to adverse developmental outcomes. Undernutrition remains a major global public health challenge among children under five years of age. According to the latest WHO, UNICEF, and World Bank Joint Child Malnutrition Estimates, approximately 150.2 million children worldwide were affected by stunting and 42.8 million by wasting in 2024. Despite progress over recent decades, the burden of undernutrition remains substantial, particularly in low- and middle-income countries, where it continues to contribute to increased morbidity, mortality, and adverse developmental outcomes [3,4]. Early childhood is a critical period for physical and neurological development, making children particularly vulnerable to the effects of poor nutrition [5].

The relationship between nutritional status and neurodevelopment in children under 5 years of age has been widely studied, particularly during the first years of life, when nutritional deficits may negatively affect motor, cognitive, and language development [6]. Neurodevelopment is a multifactorial process influenced by nutrition, genetics, and environmental stimulation [6].

The 2015 Colombian national survey on nutritional status showed that one in ten Colombian children in early childhood suffers from chronic malnutrition, accounting for 10.8% of this population [7]. In terms of overall malnutrition, it affects 3.7% of children in Colombia [7]. According to the weekly epidemiological bulletin of the Colombian Ministry of Health, 24,396 cases of children under 5 years of age with acute moderate or severe malnutrition were reported in 2024 [8]. In Bogotá, according to the document “The Nutritional Status of Early Childhood in Bogotá 2021” from the Bogotá Cómo Vamos program, chronic malnutrition has a prevalence of 12.3% in the early childhood population [9]. The reports agree on the need to strengthen prevention, identification, and control measures to reduce fatal outcomes. That is why assessing the nutritional status of young children living in vulnerable areas is not only key to prevention but also to raising awareness of the need for structural changes and the development of effective strategies that comprehensively address this problem.

Given that one of the most important factors contributing to malnutrition in children is socioeconomic status, it is essential to assess the nutritional status of children living in low-income contexts. The El Codito sector is located in the far north of the city of Bogotá, on the eastern hills. This is a highly vulnerable area where the socioeconomic conditions of its inhabitants are precarious; many families lack access to basic public services and the necessary guarantees to meet their needs, particularly those of young children [10].

Within this context, the Early Childhood Education at Home (Educación Inicial en el Hogar—EIH) program plays an important role in supporting families and promoting early childhood development in El Codito. Through nutritional support, educational activities, and family-centered interventions, the program seeks to improve child well-being in this vulnerable community.

Despite these important community-based efforts, it remains vitally important to identify the nutritional and neurodevelopmental disorders present in this sector of the city in order to adequately address the health of children and generate viable strategies aligned with the lived reality of this population. Improving nutritional status during early childhood is essential to ensure better developmental outcomes and long-term opportunities. To understand the association between nutritional status and neurodevelopment in children living in the El Codito sector, the objective of this study was to evaluate nutritional status, household food insecurity, and neurodevelopmental outcomes among children enrolled in the Early Childhood Education at Home program in El Codito and to explore the association between anthropometric indicators and neurodevelopmental performance. This evaluation will support collaborative work with families and community leaders on issues of nutrition and neurodevelopment, with the aim of contributing to the development of policies and interventions that prevent neurodevelopmental delays and improve the overall well-being of this population.

## 2. Materials and Methods

### 2.1. Study Design

A cross-sectional, quantitative, observational study was conducted to assess the nutritional status of and neurodevelopment in children under 5 years old attending the Early Childhood Education at Home program implemented in El Codito. For data collection, we built an alliance with the FUNTRAVIDI organization. Approval from the Institutional Ethics Committee was obtained. The research was conducted following the 2024 Declaration of Helsinki, the WHO ethical standards and procedures for research with human beings, and the national research regulation.

### 2.2. Participants

The target population included children under 5 years old who lived in this sector and attended the daycares associated with the foundation. The institutes selected were 18 daycares and the Estrella de Oriente school, which served approximately 400 children under 5 years. The sample size was estimated considering that the population of children under 5 years old in this sector corresponds to 8.620 people and the malnutrition prevalence in Bogota is estimated at 13%. It was calculated with a 5% margin of error, a 95% confidence level, and a statistical power of 80%, resulting in a sample size of 174 participants for this study. To preserve the statistical power of the study, a loss rate of up to 10% was considered acceptable.

The eligibility criteria included children under 5 years who attended the institutions and whose parents granted informed consent, whereas the exclusion criteria were children who were outside the age range, with identified neurological comorbidities, with extremely low weight at birth (<1000 g), who were born before 32 weeks of gestation, who had a previous diagnosis of a chronic illness, and children with conditions that may affect linear growth. Children with neurological comorbidities were excluded to minimize potential confounding in neurodevelopmental assessments.

### 2.3. Variables/Data Collection

Data was collected at the daycare centers between 9 April 2025 and 7 August 2025 by trained medical students and certified physicians affiliated with the Universidad del Rosario. The team was trained to apply the tools used and to register the data in a standardized manner.

A researcher-developed questionnaire with sociodemographic information of the family, past medical history, and current feeding pattern was applied to the parents of the included children.

Food insecurity was assessed with the Food Insecurity Experience Scale (FIES), a survey that includes eight questions to explore food access in the respondent household and has been applied at the national level in Colombia. FIES is currently used by international reference institutions such as the Food and Agriculture Organization.

Neurodevelopment assessment was carried out using the Abbreviated Developmental Scale (Escala Abreviada de Desarrollo, EAD), a developmental screening tool standardized and validated for the Colombian population by the Colombian Institute of Family Welfare (ICBF). This scale is intended for the early detection of potential neurodevelopmental delays in children through the assessment of age-appropriate developmental milestones. The EAD comprises four domains: gross motor skills, fine motor/adaptive skills, hearing and language, and personal–social skills. Each domain includes a set of age-specific activities, and scores are assigned according to the child’s ability to perform them. The instrument has demonstrated evidence of content validity, convergent validity, and reproducibility in Colombian children and is widely used in child health and development programs. However, published estimates of overall sensitivity and specificity for the complete scale are not currently available.

Subsequently, a physical examination oriented to assess nutritional status and anthropometric values was implemented. The researchers who conducted the neurodevelopmental assessments were blinded to the participants’ nutritional status. Anthropometric measurements and nutritional classification were performed independently by a nutritionist affiliated with the program, and the datasets were merged only after data collection had been completed. The measures established to assess the individual nutritional state include weight for height, height for age, body mass index for age, weight for age, and head circumference for age. All anthropometric indicators were interpreted according to WHO growth standards and expressed as Z-scores. Each indicator has an established cut-off point; they were used to establish risk and diagnosis of acute or chronic malnutrition, overweight, obesity, and or risk factor for neurodevelopment. An additional measure taken into account was the arm perimeter, which is associated with mortality due to malnutrition.

### 2.4. Statistical Analysis

Statistical analysis was performed using Jamovi version 2.6.26. Initially, a univariate analysis was conducted with the sociodemographic and clinical variables. The qualitative variables are presented in frequencies and percentages. The quantitative variables were tested to establish distribution and are reported as the mean and standard deviation or median and interquartile range. Bivariate analysis was carried out to explore the association between variables. Chi-square tests or Fisher’s exact tests were used for categorical variables, as appropriate. For quantitative variables, either Student’s *t*-test or the Mann–Whitney U test were applied depending on data distribution. Statistical significance was established with a *p*-value of less than 0.05. FIES analysis was performed according to the FAO manual recommendations. Given the exploratory nature of the study, no correction for multiple comparisons was applied, and all analyses were interpreted as individual bivariate assessments.

### 2.5. Ethics and Vulnerabilities

Parents and legal tutors received information about the purpose and procedures of the project. The ones who agreed to participate voluntarily signed an informed consent form. They could revoke their consent during any part of the study. Only the lead investigators had access to the database. The data collected was anonymized.

Furthermore, these children benefited from the assessment of their nutritional state and neurodevelopment, with individual results and recommendations provided to their caregiver. Every activity in this project was conducted in order to contribute to the well-being of the children without causing any harm nor exposing them to unnecessary risks.

## 3. Results

### 3.1. Sociodemographic Status and Perinatal Characteristics

A total of 167 patients were included in the study. The median age was 9 months (interquartile range: 5 to 15 months), with a minimum of 1 month and maximum of 4 years and 11 months. Of the patients, 53.3% (n = 89) were male. Most children did not have any ethnic affiliation (92.8%).

Regarding socioeconomic status, where levels 1 and 2 represent low income, 3 represents medium income and 4 and above represent high income, most patients were distributed in level 1 (26.9%), level 2 (39.5%), and level 3 (24.6%). Regarding residency, 65.9% of families live in an apartment. The majority of families’ homes are under rent or sublease. Most families are bi-parental nuclear (44.3%), followed by bi-parental extended (27.5%). The mean of people living in the house is 4 (IQR 3–5).

Regarding nationality, 71.3% of the children had Colombian parents, 18.6% had parents of foreign nationality, all corresponding to Venezuela, and 8.4% of parents reported having Colombian–Venezuelan nationality.

The median age of the mothers of the children was 27 years (IQR: 22.3 to 31 years). The minimum age was 16 and maximum age 46 years. Five patients had underage mothers at the time of the survey. The majority of mothers had completed their secondary education (51.5%), those who had a technical degree or higher education were 35.9%, while 12.6% had incomplete school education or no formal education at all. Regarding occupation, 53.9% were unemployed during the past year, 28.1% of mothers were employed at the time of the study, and 17.4% were unemployed but had worked during the past year.

The median age of the fathers of the children was 30.5 years (IQR: 25 to 37 years). Two patients had underage fathers at the time of the survey. Most participants reported completed secondary education (43.7%). Additionally, 28.7% had incomplete or no formal schooling, while 19.8% had attained higher education. Information was unavailable for 7.8% of the sample. A total of 83.8% of fathers were employed at the time of the survey, while 4.8% were unemployed but had worked in the past 12 months.

Median gestational age at birth was 38 weeks (IQR 37–39), with a median weight of 2940 g (IQR 2638–3156) and a median length of 49 cm (IQR 47–51 cm). From the total of children included in the study, 17.4% had past medical history of preterm birth, and 17.4% reported low birth weight. Regarding prenatal care, 85% (142 mothers) had four or more prenatal care appointments during gestation (Table 1).

During the assessment of past medical history, 78% of the children had no past medical history, while 22% of children had some past medical history, and the main diagnoses were respiratory disease, including bronchiolitis, pneumonia, asthma, or viral infections. As for neurodevelopment-related past history, one child had an epilepsy diagnosis, and two children presented abnormalities in the Auditory Evoked Potential screening (AEP) (Table 1).

The vast majority of the children (96.4%) were breastfed. Parent-reported exclusive breastfeeding persisted for a median of 6 months with an interquartile range from 4 to 6 months (Table 1).

Regarding parent-reported neurodevelopment, the median ages at which children achieved developmental milestones were as follows: head control at 2 months, sitting at 5 months, crawling at 7 months, walking at 12 months, and first words at 18 months.

### 3.2. Nutritional and Anthropometric Indicators

Anthropometric evaluations indicated that children had an average weight of 8.53 kg (SD = 2.02), height of 70.6 cm (SD = 8.36), and head circumference of 44.8 cm (SD = 3), while mid-upper arm circumference had a median of 15 cm (IQR = 14.4–16). Based on MUAC classification, 86.2% of children were within the normal range (>12.5 cm), 11.4% were at risk (11.5–12.5 cm), and 2.4% met criteria for acute malnutrition (<11.5 cm). Clinical signs of malnutrition were uncommon, with no cases of edema reported and only a small proportion presenting hair changes (3%), dry or rough skin (4.8%), hypo- or hyperpigmented spots (13.8%), visible thinness (1.8%), or palmar/mucosal pallor (1.8%). Weight for height yielded a mean Z-score of 0.17 (SD = 1.15), with 1.8% classified with acute malnutrition, 9.6% at risk, 68.3% within normal range, 17.4% at risk of overweight, and 3% classified as overweight. Height for age presented a median Z-score of −0.90 (IQR = −1.55 to 0), with 10.8% of children classified with stunting and 28.7% at risk of stunting. Weight for age had a mean Z-score of −0.38 (SD = 0.94), with 2.4% of the patients classified with global malnutrition, 19.2% at risk, and 78.4% within normal range (Table 2).

### 3.3. Neurodevelopmental Status

Children’s performance on the neurodevelopmental scale showed variable outcomes across domains. Regarding the sample’s gross motor abilities, the Z-score was −0.108 (SD 1.36); 79% (n = 132) underwent expected development for age, while 12.6% (n = 21) presented as at risk for developmental problems, and 8.4% (n = 14) had a suspected developmental delay. For fine motor development, the mean Z-score was 0.811 (SD 1.45). According to the classification system, 92.2% (n = 152) showed expected development for age, while 3% (n = 5) were classified as at risk for developmental delay, and 4.8% (n = 8) had a suspected developmental delay (Table 3).

Hearing and language neurodevelopment was presented as a Z-score of 0.1 with an interquartile range (0.65–1.2), where 83.8% (n = 140) children exhibited expected development for age in this domain, 9.6% (n = 16) were at risk for developmental delay, and 6.6% (n = 11) had a suspected developmental delay. The assessment of the personal and social neurodevelopment in this sample revealed a Z-score of 0.3 (IQR −0.3–1.3), with 89.8% (n = 150) of the children evaluated being situated within the expected neurodevelopment domain, while 4.8% (n = 8) were at risk for developmental delay, and 5.4% (n = 9) showed suspected developmental delay (Table 3).

Overall, domain-specific neurodevelopmental variability was observed. Gross and fine motor scores exhibited a normal distribution, while hearing, language, and personal–social scores showed a non-normal distribution.

### 3.4. Bivariate Analysis

#### 3.4.1. Sociodemographic and Neurodevelopment

Bivariate analyses were conducted to explore associations between sociodemographic variables and neurodevelopmental outcomes. No statistically significant differences were observed in any of the neurodevelopmental domains when comparing participants by sex, socioeconomic stratum, maternal education, paternal education, or parents’ nationality. These findings suggest that, within this sample, sociodemographic characteristics were not associated with differences in early neurodevelopmental performance (Table 4).

#### 3.4.2. Sociodemographic and Anthropometry

Bivariate analyses examining anthropometric indicators by sociodemographic variables showed mixed results. No statistically significant differences were observed in weight-for-age, weight-for-height, or height-for-age Z-scores when comparing groups by sex, maternal education, paternal education, or nationality (*p* > 0.05 for all comparisons; Table 5). However, socioeconomic status was significantly associated with height-for-age Z-scores (χ^2^(2) = 8.46, *p* = 0.015), with post hoc Dwass–Steel–Critchlow–Fligner comparisons indicating that children in socioeconomic level 1 had significantly higher height-for-age values compared with those in levels 2 (*p* = 0.033) and 3 or higher (*p* = 0.032), while no difference was observed between levels 2 and 3 or higher (*p* = 0.749). This pattern is reflected in the box plot, where the median and upper quartile for level 1 are visibly higher relative to the other categories.

In addition, paternal education demonstrated a statistically significant association with weight-for-age Z-scores (*p* = 0.038; Table 5). Children whose fathers had secondary education displayed the highest mean weight-for-age Z-score (M = −0.199), followed by those with higher education (M = −0.397), and the lowest values were observed in children whose fathers had lower educational levels (M = −0.665). Together, these findings suggest that socioeconomic status and paternal education may play a role in children’s growth patterns within this sample, whereas other sociodemographic characteristics did not demonstrate significant associations with anthropometric outcomes.

#### 3.4.3. Neurodevelopment and Anthropometry

Moderate positive correlations were observed among the four neurodevelopmental domains. Gross motor scores correlated significantly with fine motor performance: ρ = 0.451, *p* < 0.001; audition–language scores: ρ = 0.358, *p* < 0.001; and personal–social scores: ρ = 0.418, *p* < 0.001. Fine motor scores were also significantly correlated with audition–language: ρ = 0.434, *p* < 0.001; and personal–social performance: ρ = 0.435, *p* < 0.001. Finally, audition–language and personal–social domains were strongly correlated: ρ = 0.535, *p* < 0.001.

Collectively, these results indicate a coherent pattern of inter-related neurodevelopmental skills and small but significant relationships between specific anthropometric indicators and neurodevelopmental outcomes.

Regarding the association between anthropometric indicators and neurodevelopmental performance, a weak but statistically significant positive correlation was found between weight-for-age Z-score (W/A) and gross motor scores: r = 0.153 (95% CI: 0.001–0.298), *p* = 0.048. Likewise, W/A showed a significant positive correlation with fine motor scores (r = 0.163 (95% CI: 0.011–0.307), *p* = 0.036), as well as with audition–language scores (ρ = 0.155, *p* = 0.046).

Regarding weight-for-length, the weight-for-length Z-score (W/L) was positively correlated with gross motor scores: r = 0.204 (95% CI: 0.053–0.345), *p* = 0.008. No significant correlations were observed between W/L and fine motor, audition–language, or personal–social domains. For height-for-age Z-score (H/A), no significant associations were identified with any neurodevelopmental domain (all *p* > 0.40).

## 4. Discussion

Exploring how nutrition and neurodevelopment interact can offer useful insights for designing programs and policies that promote healthy early childhood development. This study included children under five years old living in a vulnerable sector of Bogotá characterized by socioeconomic disadvantage. The median age of participants was 9 months, with a slightly higher proportion of males (53.3%). These structural vulnerabilities are important to consider, as they are known to influence both nutritional status and developmental outcomes [11,12,13].

In terms of nutritional status, anthropometric assessment showed a dual pattern of nutritional risk. Although the prevalence of acute malnutrition was low (1.8%), 28.7% of children were at risk of stunting, and 10.8% met criteria for stunting. These findings align with global and Latin American evidence demonstrating the persistence of linear growth faltering in socioeconomically vulnerable populations [12,13,14,15,16]. Weight-for-age Z-scores indicated that 19.2% of children were at risk of global malnutrition. Additionally, 17.4% were at risk of overweight and 3% were classified as overweight, reflecting the emergence of the double burden of malnutrition described in other low-income countries [11,17,18].

Breastfeeding rates were high, with 96.4% of children reported to have received breast milk, and the median duration of exclusive breastfeeding was 6 months. These adequate breastfeeding practices may help buffer some of the developmental risks usually linked to early nutritional vulnerability [19,20].

Most children demonstrated age-appropriate neurodevelopmental performance across all domains of the EAD, suggesting relative developmental resilience despite the socioeconomic vulnerabilities present in the community, being consistent with the literature [17,21].

Importantly, moderate positive correlations were observed among neurodevelopmental domains. Gross motor scores showed significant associations with fine motor (ρ = 0.451), hearing–language (ρ = 0.358), and personal–social skills (ρ = 0.418). Hearing–language and personal–social domains also displayed a strong correlation (ρ = 0.535), and similar inter-domain relationships have been widely documented in early childhood research, emphasizing the integrated nature of motor, cognitive, and social developmental processes [21,22,23,24].

### 4.1. Anthropometry and Neurodevelopment

Small but statistically significant associations were identified between anthropometric measures and developmental performance. Weight for age was positively correlated with gross motor (r = 0.153), fine motor (r = 0.163), and hearing–language (ρ = 0.155) scores. Although these correlations reached statistical significance, their magnitude was relatively small and should therefore be interpreted as population-level associations rather than strong predictive relationships at the individual level. These associations are consistent with evidence showing that insufficient weight gain can affect neuromuscular development and early cognitive–language processes, possibly through mechanisms such as altered myelination or reduced neural energy availability [20,21,25,26]. Adequate nutrition during infancy supports rapid brain growth, synaptic development, and motor maturation, processes that may influence a child’s ability to interact with and learn from their environment. The association observed across motor and language domains supports the concept that nutritional status and neurodevelopment are closely interconnected processes during early childhood, even when the magnitude of the observed effects is modest. Weight for length was significantly associated only with gross motor development (r = 0.204), which aligns with studies indicating that acute nutritional deficits may affect muscle mass and strength before influencing higher-order cognitive skills [11,17].

Height for age did not correlate significantly with any neurodevelopmental domain. Although stunting is traditionally associated with global developmental delays [12,25], the absence of association in this sample may reflect the young median age, protective factors, or limited variability in linear growth. Additionally, children enrolled in this study participated in an early childhood program implemented by FUNTRAVIDI that provided nutritional counseling and monitoring, occupational therapy, speech and language support, and psychological follow-up, which may have contributed to supporting developmental outcomes despite the presence of nutritional risk. Similar findings have been reported in early-infancy cohorts, where the neurodevelopmental effects of stunting become more apparent after two years of age [17,25], suggesting that the consequences of chronic undernutrition may not yet be fully manifested in a predominantly infant population.

### 4.2. Sociodemographic Variables and Development

No significant associations were found between neurodevelopmental outcomes and sex, socioeconomic status, parental education, or nationality. This differs from global evidence showing strong links between environmental disadvantage and developmental trajectories [21]. However, the relative homogeneity of socioeconomic hardship in this cohort may have reduced variability, making it harder to detect these relationships statistically. Additionally, this population was exposed to various protective factors that reduce differences between groups, while participation in structured early childhood programs like the one implemented in this study may have further supported nutritional status and neurodevelopment, potentially buffering the effects of socioeconomic disadvantage [17,19].

In contrast, socioeconomic status was significantly associated with height for age, while paternal education was associated with weight for age. Previous studies conducted in low- and middle-income countries have shown that paternal education may influence child growth and development through its effects on household resources, caregiving practices, and access to health-related opportunities [24]. Interestingly, children from the lowest socioeconomic stratum in our cohort presented higher height-for-age values than those from higher strata. This finding differs from most epidemiological evidence, which generally reports poorer linear growth among children living in more disadvantaged socioeconomic conditions and identifies stunting as a marker of broader social and nutritional vulnerability [25]. One possible explanation is that the socioeconomic categories within this community may not represent differences as marked as those observed in population-based studies, given that most participating families shared similar conditions of social vulnerability. Additionally, all children were enrolled in the same early childhood program and had access to nutritional monitoring and developmental support, which may have reduced some of the disparities typically associated with socioeconomic status and should be considered when interpreting this finding.

The estimated prevalence of moderate-to-severe food insecurity was 21.4% (±9.3), while severe food insecurity reached 2.6% (±3.2). These estimates are comparable to results from the National Quality of Life Survey 2023–2024, which applied the FIES questionnaire. The reported prevalence of moderate-severe food insecurity was 25.5%, and severe food insecurity was 5.0% nationwide in 2024. In households with at least one child under 5 years old, the prevalence was estimated at 31.5% in 2024. Bogotá reported a prevalence of moderate–severe food insecurity of 13.9% and severe food insecurity of 2.8% during the same year. However, these findings should be interpreted within the context of an early childhood program that provided nutritional counseling and support to participating families, which may have mitigated some of the effects of food insecurity on nutritional and developmental outcomes. Food security remains a key component of child well-being, as it influences not only access to adequate nutrition but also the stability of the environment in which children grow and develop.

## 5. Conclusions

This study highlights a considerable prevalence of nutritional risk among young children living in a socioeconomically vulnerable area of Bogotá, particularly regarding stunting and risk of stunting. Although most children demonstrated age-appropriate neurodevelopment, the identification of small but meaningful associations between weight for age and several developmental domains reinforces the importance of adequate early-life nutrition for motor, cognitive, and language development. These findings are consistent with global evidence emphasizing the close relationship between physical growth and neurodevelopment during early childhood.

The results suggest the need to strengthen early childhood programs that integrate nutritional assessment, developmental screening, and caregiver education, especially in underserved communities. Interventions aimed at improving growth trajectories—such as promoting optimal feeding practices, improving food security, and encouraging early stimulation—may help reduce long-term developmental gaps. Ongoing research and coordinated public health efforts will be key to addressing structural barriers, reducing inequalities, and supporting children’s developmental potential in similar high-vulnerability settings.

## 6. Limitations of the Study

This study has several limitations that should be considered when interpreting the findings. The relative socioeconomic homogeneity of the cohort may have reduced variability across exposure groups, limiting the ability to detect potential associations. The modest sample size and observational design also increase the possibility of residual confounding and limit causal inference. In addition, no adjustment for multiple comparisons was applied because of the exploratory nature of the analyses, which may have increased the risk of type I error. Given the cross-sectional nature of the study, the observed associations should be interpreted as correlational and do not permit conclusions about causality. In addition, selection bias cannot be excluded, as families who remained engaged in follow-up and early childhood services may differ systematically from those not represented. Future research could help address these points by including more diverse and larger samples and adopting longitudinal approaches.

## Figures and Tables

**Table 1:** Background.

Gestational Age	38 Weeks (37–39)	
Birth weight	2940 g (2638–3156)	
Birth length	49 cm (47–51)	
Low birth weight	Yes	17.4% (29)
	No	82.6% (138)
Preterm birth	Yes	17.4% (29)
	No	82.6% (138)
≥4 prenatal visits	Yes	85% (142)
	No	15% (25)
Other perinatal history	Yes	38.9% (65)
(high obstetric risk, neonatal unit admission, etc.)	No	61.1% (102)
Past medical history	Yes	22% (37)
	No	78% (130)
Breastfed	Yes	96.4% (161)
	No	1.8% (3)
	No information	1.8% (3)
Duration of exclusive breastfeeding (months)	6 months (IQR 4–6)	

**Table 2:** Nutritional status.

**Weight for Height**		
Z-score	0.174 (SD 1.15)	
Category	Acute malnutrition	1.8% (3)
	At risk of acute malnutrition	9.6% (16)
	Normal weight for height	68.3% (114)
	At risk of overweight	17.4% (29)
	Overweight	3% (5)
**Height for Age**		
Z-score	−0.9 (RIQ −1.55–0)	
Category	Stunting	10.8% (18)
	At risk of stunting	28.7% (48)
	Normal height for age	60.5% (101)
**Weight for Age**		
Z-score	−0.381 (DE 0.936)	
Category	Global malnutrition	2.4% (4)
	At risk of global malnutrition	19.2% (32)
	Normal weight for age	78.4% (131)

**Table 3:** Neurodevelopmental scale.

**Motor gross neurodevelopment**		
Z-score	−0.108 (SD 1.36)	
Classification	Expected development for age	79% (132)
	Risk for developmental delay	12.6% (21)
	Suspected developmental delay	8.4% (14)
**Fine motor neurodevelopment**		
Z-score	0.811 (SD 1.45)	
Classification	Expected development for age	92.2% (154)
	Risk for developmental delay	3% (5)
	Suspected developmental delay	4.8% (8)
**Hearing and language neurodevelopment**		
Z-score	0.1 (IQR −0.65–1.2)	
Classification	Expected development for age	83.8% (140)
	Risk for developmental delay	9.6% (16)
	Suspected developmental delay	6.6% (11)
**Personal and social neurodevelopment**		
Z-score	0.3 (IQR −0.3–1.3)	
Classification	Expected development for age	89.8% (150)
	Risk for developmental delay	4.8% (8)
	Suspected developmental delay	5.4% (9)

**Table 4:** Neurodevelopment according to sex, socioeconomic status, mother’s education, father’s education, and nationality.

	Sex	Socioeconomic Status	Mother’s Education	Father’s Education	Nationality
Neurodevelopmental Score	*p*-Value	*p*-Value	*p*-Value	*p*-Value	*p*-Value
Gross motor	0.834	0.466	0.833	0.910	0.163
Fine motor	0.224	0.332	0.338	0.294	0.437
Language and hearing	0.283	0.129	0.079	0.725	0.669
Personal–social	0.709	0.970	0.553	0.600	0.511

**Table 5:** Anthropometric data according to sex, socioeconomic status, mother’s education, father’s education, and nationality.

	Sex	Socioeconomic Status	Mother’s Education	Father’s Education	Nationality
Z-Score	*p*-Value	*p*-Value	*p*-Value	*p*-Value	*p*-Value
Weight/age	0.504	0.224	0.405	0.038	0.834
Weight/height	0.134	0.336	0.746	0.324	0.545
Height/age	0.25	0.015	0.639	0.690	0.490

## Data Availability

The data is not publicly available due to ethical restrictions and the inclusion of sensitive information from minors.

## References

[1] UNICEF Spain (2011). Child Malnutrition: Causes, Consequences and Strategies for Prevention and Treatment.

[2] World Health Organization (2024). Malnutrition: Fact Sheet.

[3] Black M.M. (2003). Micronutrient deficiencies and cognitive functioning. J. Nutr..

[4] Instituto Nacional de Salud (2024). Surveillance Protocol for Acute Malnutrition in Children Under 5 Years.

[5] Ministerio de Salud y Protección Social (2021). Health Situation Analysis (ASIS) Colombia 2021.

[6] Luna Hernández J.A., Hernández Arteaga I., Rojas Zapata A.F., Cadena Chala M.C. (2018). Nutritional status and neurodevelopment in early childhood. Rev. Cuba. Salud Pública.

[7] Colombian Institute of Family Welfare (2016). National Survey of Nutritional Status (ENSIN).

[8] Instituto Nacional de Salud (2025). Epidemiological Bulletin–Week 11 (2025).

[9] Bogotá Cómo Vamos (2021). Nutritional Situation in Early Childhood.

[10] Guevara Salamanca J.D., Hernández García M., Mendoza Molina M.A. (2013). Construction and Meaning of Territory: El Codito Community.

[11] Black R.E., Victora C.G., Walker S.P., Bhutta Z.A., Christian P., de Onis M., Ezzati M., Grantham-McGregor S., Katz J., Martorell R. (2013). Maternal and child undernutrition and overweight in low-income and middle-income countries. Lancet.

[12] Victora C.G., Christian P., Vidaletti L.P., Gatica-Domínguez G., Menon P., Black R.E. (2021). Revisiting maternal and child undernutrition in low-income and middle-income countries: Variable progress towards an unfinished agenda. Lancet.

[13] Santa-Ramírez H.A., Otálvaro-Castro G.J., Joost S., Melgar-Quiñonez H., Bilal U., Stringhini S. (2023). Small area vulnerability, household food insecurity and child malnutrition in Medellin, Colombia: Results from a repeated cross-sectional study. Lancet Reg. Health Am..

[14] Alvarez Morán J.L., Alé F.G., Rogers E., Guerrero S. (2018). Quality of care for treatment of uncomplicated severe acute malnutrition delivered by community health workers in a rural area of Mali. Matern. Child Nutr..

[15] Monteiro C.A., Benicio M.H.D.A., Conde W.L., Konno S.C., Lovadino A.L., Barros A.J.D., Victora C.G. (2010). Narrowing socioeconomic inequality in child stunting: The Brazilian experience, 1974–2007. Bull. World Health Organ..

[16] Benjamin-Chung J., Mertens A., Colford J.M., Hubbard A.E., van der Laan M.J., Coyle J., Sofrygin O., Cai W., Nguyen A., Pokpongkiat N.N. (2023). Early-childhood linear growth faltering in low- and middle-income countries. Nature.

[17] Grantham-McGregor S., Cheung Y.B., Cueto S., Glewwe P., Richter L., Strupp B., International Child Development Steering Group (2007). Developmental potential in the first 5 years for children in developing countries. Lancet.

[18] Liang R., Goto R., Okubo Y., Rehkopf D.H., Inoue K. (2025). Poverty and childhood obesity: Current evidence and methodologies for future research. Curr. Obes. Rep..

[19] Georgieff M.K. (2017). Nutrition and the developing brain: Nutrient priorities and measurement. Am. J. Clin. Nutr..

[20] Prado E.L., Dewey K.G. (2014). Nutrition and brain development in early life. Nutr. Rev..

[21] Black M.M., Walker S.P., Fernald L.C.H., Andersen C.T., DiGirolamo A.M., Lu C., McCoy D.C., Fink G., Shawar Y.R., Shiffman J. (2017). Early childhood development coming of age: Science through the life course. Lancet.

[22] Libertus K., Violi D.A. (2016). Sit to talk: Relation between motor skills and language development in infancy. Front. Psychol..

[23] Iverson J.M. (2010). Developing language in a developing body: The relationship between motor development and language development. J. Child Lang..

[24] Jeong J., McCoy D.C., Fink G. (2017). Pathways between paternal education and early child development in 44 low- and middle-income countries. Early Child. Res. Q..

[25] Sudfeld C.R., McCoy D.C., Danaei G., Fink G., Ezzati M., Andrews K.G., Fawzi W.W. (2015). Linear growth and child development in low- and middle-income countries: A meta-analysis. Pediatrics.

[26] Dipasquale V., Cucinotta U., Romano C. (2020). Acute Malnutrition in Children: Pathophysiology, Clinical Effects and Treatment. Nutrients.

